# Purification and biochemical characterization of a newly produced yellow laccase from *Lentinus squarrosulus* MR13

**DOI:** 10.1007/s13205-014-0219-8

**Published:** 2014-05-11

**Authors:** Mainak Mukhopadhyay, Rintu Banerjee

**Affiliations:** Microbial Biotechnology and Downstream Processing Laboratory, Agricultural and Food Engineering Department, Indian Institute of Technology, Kharagpur, 721 302 India

**Keywords:** *Lentinus squarrosulus* MR13, Yellow laccase, Circular dichroism spectroscopy, SDS-PAGE, Enzyme kinetics

## Abstract

A novel yellow laccase was produced from *Lentinus squarrosulus* MR13 under solid state fermentation. The yellow laccase was purified by a factor of 12.67-fold by ammonium sulfate precipitation, anion exchange chromatography and gel filtration chromatography to a specific activity of 3,772.86 IU mg^−1^. Its molecular mass was determined by SDS-PAGE and found to be 66 kDa. The activity of the enzyme was measured with 2,2′-azinobis-(3-ethylbenzothiazoline-6-sulfonic acid) as substrate and found to be stable in a broad range of pH (pH 4–9). The optimum temperature of the enzyme was 40 °C. The enzyme was stable at temperatures between 25 and 55 °C and decreased rapidly when the temperature was above 65 °C. Circular dichroism spectra also supported the temperature stability of the enzyme. The *K*_m_ and *V*_max_ values of the purified yellow laccase were 0.0714 mM and 0.0091 mM min^−1^, respectively.

## Introduction

Laccase was first identified and reported by Yoshida (1883) from *Rhus vernicifera*, the Japanese lacquer tree. It was characterized as a copper-containing oxidase by Bertrand (1985). Fungal laccases possess various physiological roles. Laccases from the white-rot fungi, e.g. *Trametes versicolor* and *Pycnoporus cinnabarinus*, contribute to lignin biodegradation by oxidizing the phenolic subunits of lignin (Bourbonnais et al. 1995; Eggert et al. 1996; Thurston 1994). This multipurpose enzyme is used for various purposes which include dye decolorization (Abadulla et al. 2000), fruit juice stabilization (Minussi et al. 2002), pulp bioleaching (Arias et al. 2003) and phenol and aromatic compounds degradation (Crecchio et al. 1995). In fungal physiology, laccases are involved in plant pathogenesis, pigmentation, detoxification and lignin degradation.

Yellow laccase isolated from *Panus tigrinus* 8/18 could oxidize veratryl alcohol and nonphenolic lignin as model compounds without any synthetic mediator in the reaction mixture. Yellow laccase produced by different fungi did not show the characteristic absorption spectrum at 610 nm which is a typical characteristic feature of blue laccase due to the presence of type-I copper atom. The EPR and circular dichroism (CD) spectrum are also different for this group of laccases (Leontievsky et al. 1997; Rodakiewicz-Nowak et al. 1999). Yellow laccase produced during submerged fermentation is capable of oxidizing non-phenolic compounds in the absence of any mediators. This which is a unique characteristic that differentiates yellow laccases from blue laccase (Leontievsky et al. 1997). Information on the purification and characteristics of yellow laccases is extremely limited and their catalytic properties are seldom investigated or reported. Information on the catalytic properties of these enzymes could serve as a basis for the development of bio-preparations and creation of an effective technology for their application in bioremediation (Bezalel et al. 1996) and delignification of potential lignocellulosic substrate for biofuel generation (Baldrian 2004).

In this present investigation, a new yellow laccase from *Lentinus squarrosulus* MR13 was purified and characterized biochemically. The fundamental characteristics of the purified enzyme such as molecular mass, effect of pH, temperature on the enzyme activity and stability, isoelectric point, effect of different additives and inhibitors on enzyme activity were also studied.

## Materials and methods

### Chemicals

2,2′-Azinobis-(3-ethylbenzothiazoline-6-sulfonic acid) (ABTS) was purchased from Sigma-Aldrich Company (USA). Other chemicals were of reagent grade.

### Microorganisms and enzyme production conditions

*Lentinus squarrosulus* (MR13) was collected from decayed wood in local field at Kharagpur and maintained in potato dextrose agar. Sub culturing was done in every 15 days to maintain its viability.

Substrate (rice straw) for the yellow laccase production (particle size 1 cm) was taken and Czapek-dox media was added to it in the ratio of 1:3. The substrate was mixed thoroughly, sterilized and inoculated with the inoculum prepared on wheat grain. Fermentation was carried out at 30 °C for 9 days.

After incubation the fermented biomass was soaked with water in 1:1 for 4 h at room temperature (32 °C) for leaching of the extracellular enzyme. Extraction of the enzyme from the biomass was performed using cotton cheese cloth. Maximum amount of extract was collected by extraction with pressure. The extract was centrifuged at 10,000 rpm at 4 °C for 10 min. The clear supernatant was used for subsequent studies.

### Enzyme assay

Laccase activity was determined spectrophotometrically with ABTS as substrate and the oxidation was monitored at 436 nm (*ε* = 29,300 M^−1^ cm^−1^). One unit of enzyme activity (IU) is defined as the amount of enzyme that released 1 µmol of oxidized product per minute.

### Protein estimation

The protein content of the culture filtrate was estimated by Lowry’s method (Lowry et al. 1951) with bovine serum albumin as the internal standard.

### Enzyme purification

Crude enzyme broth was concentrated by ammonium sulfate precipitation. The precipitated enzyme was dialysed and monitored at 280 nm followed by activity assay. The concentrated enzyme was loaded into a DEAE cellulose column (10 mm × 40 mm). The purified fraction was further subjected to Sephadex G 100. The final fraction of enzyme was further concentrated with Amicon ultrafiltration system having 10 kDa molecular mass cut off membrane. The purified enzyme was subsequently used for further characterization. SDS-PAGE was carried out to determine the homogeneity and apparent molecular mass of the purified laccase.

### Determination of isoelectric point

Isoelectric pH of the purified laccase was determined using Rotofor (BioRad). Focusing was carried out at a constant power of 12 W for 2 h. Twenty samples were collected and analyzed for enzyme activity and pH. The ampholyte used was in the pH range of 3.0–10.0.

### UV/visible absorption studies

Enzyme preparation was adjusted to 0.5 mg mL^−1^ in 0.1 M phosphate buffer (pH-6.8). The ratio *A*_280_/*A*_610_ was used for the characterization of laccase.

### Effect of pH and temperature on yellow laccase activity and stability

To estimate the optimum pH value, the activity of the purified enzyme was studied over a pH range of 3.0–10. For the determination of the pH stability, enzyme was kept at 4 °C for 1 h in different buffers (0.1 M) and the residual yellow laccase activity determined under standard assay conditions.

Effect of different temperatures on yellow laccase activity was measured at 25–65 °C by standard enzyme assay. To evaluate the temperature effect on the stability of yellow laccase, 100 µL protein solution was prepared in 0.1 M phosphate buffer of pH 6.8, kept at different temperatures ranging 25–65 °C for 60 h and the residual activity tested by standard enzyme assay measured.

### Circular dichroism (CD) spectra of yellow laccase

CD spectra of the purified laccase was recorded at various temperature ranging 30**–**65 °C in 0.1 M sodium phosphate buffer (pH 6.8) using a CD Spectropolarimeter (JASCO J**–**810) (Yang et al. 1986).

### Effect of metal ions on yellow laccase activity

To study the effect of various metal ions on enzyme activity, the enzyme was incubated with 1 mM of CaCl_2_, CuSO_4_, CoCl_2_, CuCl_2_, (NH_4_)_2_SO_4_,_·_FeSO_4_, FeCl_3_, FeCl_2_, K_2_SO_4_, NiSO_4_, KCl, MnCl_2_, MnSO_4_, NaCl, MgSO_4_, Na_2_SO_4_, Na_2_S, MgCl_2_, Hg_2_Cl_2_ and HgCl_2_ at 35 °C for 1 h. Then the required volume of enzyme was taken for assay by standard enzyme assay protocol.

### Effect of inhibitors and different additives

Six potential laccase inhibitors were selected to evaluate their effect on the purified laccase. The enzyme was incubated with various inhibitors for 1 h at 35 °C and laccase activity was measured.

Effect of additives like sodium azide, dithiothreitol, l-cysteine, EDTA, sodium thioglycolate, β-mercaptoethanol, SDS, urea and hydrogen peroxide on activity of purified enzyme was thereafter evaluated. Enzyme solution was incubated with different concentrations of β-mercaptoethanol, SDS and urea for 1 h at 35 °C and enzyme activities were calculated.

### Assay of substrate specificity and enzyme kinetics of yellow laccase

For the determination of substrate specificity of the enzyme, different aromatic substrates were added to the assay media by replacing ABTS. The substrates used were resorcinol, gallic acid, catechol, pyrogallol, syringaldazine, ferulic acid, vanillic acid and caffeic acid at 1 mM concentration during the assay. The substrate oxidation rate was followed by measuring the absorbance change with the molar extinction coefficient (*ε*) obtained from the literature (Wolfenden and Wilson 1982). Relative activity of ABTS was taken as 100 %.

Kinetic constants of the enzyme for the most common substrate ABTS were determined. The rate of substrate oxidation was determined by spectrophotometric method, using reported molar extinction coefficient *ε* (29,300 M^−1^ cm^−1^) (Wolfenden et al. 1982). Concentration range of ABTS was 40–600 mM. Reactions were conducted at 35 °C. *K*_m_ and *V*_max_ values were calculated using the Lineweaver–Burk plot of the Michaelis–Menten equation.

### Application of yellow laccase on lignocellulosic substrate for lignin degradation

*Bambusa bamboos* was selected for the application of yellow laccase. It is a potential substrate for the production of second generation bioethanol. To achieve higher accessibility in the cellulosic layer of the lignocellulosics, lignin must be degraded by pre-treatment process. In this experiment, crude yellow laccase was used to degrade lignin. After each cycle of the incubation of lignocellulosics with enzyme the residual lignin was estimated (Hussain et al. 2002).

## Results

### Purification of yellow laccase

**Fig. 1:**
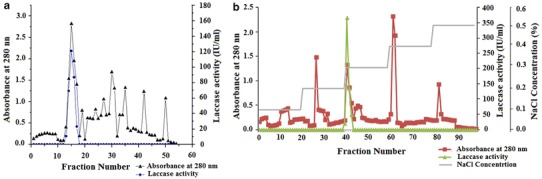
**a** Ion exchange, **b** gel filtration chromatography profile of yellow laccase

The crude extract of the yellow laccase produced from *L. squarrosulus* MR13 was purified to homogeneity using a three-step purification procedure as summarized in Table 1. The first step of ammonium sulfate precipitation (60 %) resulted in an increase in specific activity to 827.27 IU mg^−1^ protein having 2.77-fold purification. The concentrated fraction was further subjected to ion exchange chromatography where a 7.43-fold increase in purification was achieved (Fig. 1a). The eluted fraction from ion exchange chromatography was pooled and used for gel filtration chromatography. The specific activity of laccase was found to be further increased to 3,772.86 IU mg^−1^ protein having 12.67-fold purification (Fig. 1b).

**Table 1:** Purification profile of yellow laccase of *Lentinus squarrosulus* MR13 produced by solid state fermentation

Purification step	Protein concentration (mg mL^−1^)	Enzyme activity (IU mL^−1^)	Specific activity (IU mg^−1^)	Purification (fold)	% Yield
Crude extract	1.985	593.8	299.14	01.00	100
(NH_4_)_2_SO_4_ precipitation	2.515	2,080.6	827.27	02.77	81
DEAE cellulose	0.062	137.83	2,223.06	07.43	64
Sephadex G 100	0.021	79.23	3,772.86	12.61	45

### Determination of molecular mass

**Fig. 2:**
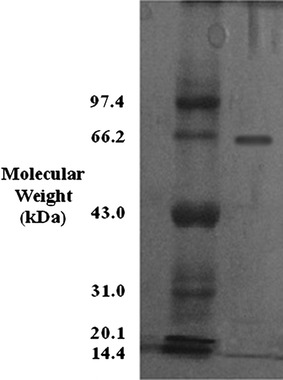
SDS-PAGE for determination of purity and calculation of molecular weight of the yellow laccase (molecular weight 66 kDa)

The purified *L. squarrosulus* MR13 yellow laccase showed a single band on SDS-PAGE with a mobility equivalent to the molecular mass of 66 kDa as visualized by Coomassie brilliant blue staining (Fig. 2).

### UV/visible absorption studies

**Fig. 3:**
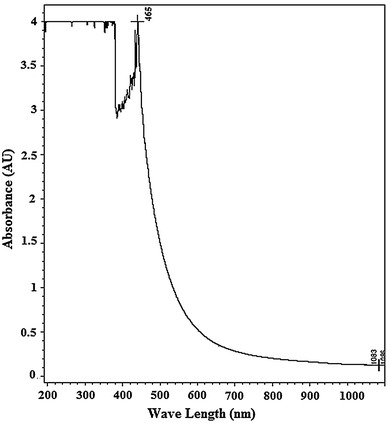
UV/vis absorption spectra of purified yellow laccase

Absorption maxima of the purified enzyme were observed at 465 nm (Fig. 3). The absorbance spectra of purified enzyme showed no significance absorbance at 610 nm. The *A*_280_/*A*_610_ ratio of purified enzyme was 312.5. This value indicated the absence of type III copper atom in the purified enzyme. From this observation it was concluded that the enzyme belongs to yellow laccase.

### Determination of p*I*

Isoelectric focusing is an extremely powerful, non denaturing technique for localization of the proteins at their respective isoelectric points without any phase change. The absence of any phase change, separation of proteins in their native state and efficient temperature control in Rotofor^®^ system leads to minimum denaturation of the enzyme. Isoelectric focusing of laccase revealed that there are only one laccase present which had p*I* at pH 8.2.

### Effect of pH on yellow laccase activity and stability

**Fig. 4:**
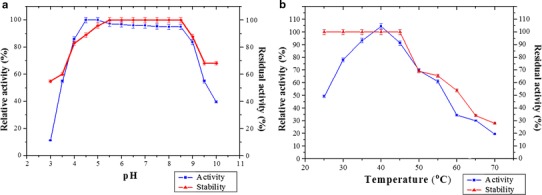
**a** Effect of pH and **b** temperature on the activity and stability of purified yellow laccase

The effect of pH on enzyme activity was analyzed by carrying out enzyme assays at different pH. The highest activity was observed at pH 4.5. Enzyme was able to hold back its activity in a long range of pH, from 4.0 to 9.0 (Fig. 4a). At pH 3.5 and 9.5 enzyme was able to retain its half activity. The purified enzyme exhibits maximum stability at the pH range 5.0–8.5. Comparable stability was also observed at pH 4.0, 4.5 and 9.0.

### Effect of temperature on yellow laccase activity and stability

The optimum temperature of laccase activity was obtained at 40 °C, beyond which the activity of the enzyme started decreasing (Fig. 4b). From the obtained results the profile of enzyme activity can be shown as 35 > 40 > 45 > 30 > 50 > 55 > 60 > 65 °C. Results for the effect of temperature on stability of yellow laccase indicated that the enzyme under evaluation has been considered to be stable in moderate to higher temperature. It could withstand the temperature up to 55 °C, but at 65 °C its activity was reduced to 34 %.

### CD spectra of yellow laccase at different temperature

**Fig. 5:**
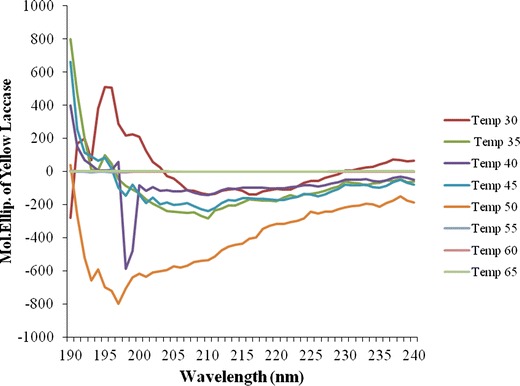
CD spectra of yellow laccase at different temperature

At lower temperature, yellow laccase showed more α-helix and minimum random coil. At higher temperature percentage of α-helix has decreased, which indicated that yellow laccase was more active at lower to moderate temperature (Fig. 5). In the temperature range 30–50 °C yellow laccase showed almost equivalent amount of α helix than β sheet (Table 2), which indicated the normal activity of yellow laccase. The spectral features of the enzyme at the temperature range of 30–50 °C remained almost constant with slight variations in magnitude.

**Table 2:** α Helix and β sheet comparison of yellow laccase at different temperature

Temperature (°C)	α Helix (%)	β Sheet (%)
30	67.84	9.46
35	69.24	8.63
40	70.50	8.40
45	69.48	8.63
50	62.57	9.60
55	4.23	29.74
60	3.80	31.70
65	1.53	33.80

### Effect of metal ions

The stability of enzyme activity against different metal ions was studied and it was observed that the yellow laccase retains its normal activity in the presence of (NH_4_)_2_SO_4_, whereas the activity was increased ~2 % in presence of CuCl_2_ and 5 % in presence of CuSO_4_. All the other metal ions acted as the inhibitor of the enzyme in 1 mM concentration (Table 3).

**Table 3:** Effect of metal ions on the activity of the purified yellow laccase from *Lentinus squarrosulus* (MR13)

Metal ions (1 mM)	Relative activity (%)
Control	100.00
CaCl_2_	84.82
CuSO_4_	105.78
CoCl_2_	90.57
(NH_4_)_2_SO_4_	100.22
FeCl_3_	72.70
FeCl_2_	89.13
K_2_SO_4_	97.35
NiSO_4_	98.99
KCl	95.91
MnCl_2_	89.34
MnSO_4_	93.04
NaCl	83.79
MgSO_4_	88.31
Na_2_SO_4_	90.78
Na_2_S	10.27
Hg_2_Cl_2_	44.57
HgCl_2_	61.82

### Effect of inhibitors

Effect of different inhibitors on enzyme activity was observed using sodium azide, dithiothreitol, l-cysteine, EDTA, sodium thioglycolate and H_2_O_2_ (Table 4). It was observed that 1 mM concentration of sodium azide, sodium thioglycolate and l-cysteine completely inhibited the enzyme activity. EDTA and H_2_O_2_ were able to inhibit the enzyme completely in higher concentrations, 2 and 5 mM, respectively. Dithiothreitol acted as a potent inhibitor which was able to inhibit the enzyme completely at 0.1 mM concentration.

**Table 4:** Effect of Inhibitors on purified yellow laccase from *Lentinus squarrosulus* (MR13)

Inhibitors	Concentration (mM)	Inhibition (%)
Control (no inhibitor)	–	0.00
Sodium azide	0.1	33.33
	1.0	100.00
Dithiothreitol	0.1	100.00
l-Cysteine	0.1	32.22
	1.0	100.00
EDTA	0.1	6.67
	1.0	72.38
	5.0	100.00
Sodium thioglycolate	0.1	58.64
	1.0	100.00
H_2_O_2_	0.1	57.00
	1.0	84.63
	2.0	100.00

### Effect of different additives

The effect of additives like β-mercaptoethanol, SDS and urea, on activity of purified enzyme was evaluated. Results exhibited that β-mercaptoethanol, SDS and urea at their higher concentration were to inhibit the enzyme completely (Table 5). β-mercaptoethanol and urea at 0.1 mM concentration were able to inhibit only 14.29 and 12.5 % of enzyme activity, respectively. At 10 mM concentration of SDS the enzyme was completely inhibited.

**Table 5:** Effect of different additives on purified yellow laccase from *Lentinus squarrosulus* (MR13)

Additives	Concentration (mM)	Inhibition (%)
Control	–	0.00
β-Mercaptoethanol	0.1	14.29
	1.0	42.86
	10.0	67.86
	100.0	100.00
SDS	0.01	7.14
	0.1	31.67
	1.0	69.64
	10.0	100.00
Urea	0.1	12.50
	1.0	20.28
	5.0	55.19
	10.0	100.00

### Substrate specificity

The data on substrate specificity of the enzyme are summarized in Table 6. Syringaldazine showed highest relative activity (116.69 %), whereas other substrates like resorcinol, catechol, tannic acid, ferulic acid, vanillic acid and caffeic acid have nearly similar specificity towards the purified yellow laccase.

**Table 6:** Substrate specificity of purified yellow laccase from *Lentinus squarrosulus* (MR13)

Substrate (1 mM)	Wavelength (nm)	Absorption coefficient (M^−1^ cm^−1^)	Relative activity (%)
ABTS	436	29,300	100.00
Resorcinol	500	32,800	73.68
Gallic acid	250	4,910	78.29
Catechol	450	2,211	81.65
Pyrogallol	450	4,400	53.23
Syringaldazine	530	65,000	116.87
Ferulic acid	287	12,483	84.92
Vanillic acid	316	2,340	83.98
Caffeic acid	311	18,500	85.69

### Kinetic properties of purified yellow laccase

ABTS was used as the substrate to determined two main kinetic parameters *V*_max_ and *K*_m_. Lineweaver–Burk plot confirmed that the *K*_m_ and *V*_max_ values of the purified yellow laccase were 0.0714 mM and 0.0091 mM min^−1^. The *K*_cat_ of the purified yellow laccase was found to be 303 s^−1^. Catalytic efficiency (*K*_cat_/*K*_m_) of the enzyme was found as 4,089 s^−1^ mM^−1^.

### Lignin degradation studies of lignocellulosic substrate

**Fig. 6:**
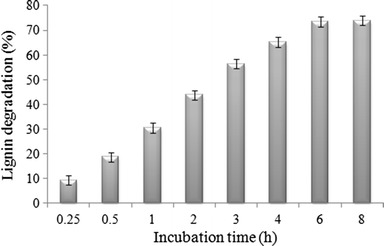
Lignin degradation profile of yellow laccase in *Bambusa bamboos* against time

The capability of white rot fungi to degrade polyaromatic compounds, dyes and lignin has been widely studied. In this present work, crude yellow laccase was used to degrade the lignin from *B. bamboos*. The degradation studies were conducted at the conditions most suitable for the enzymatic action. Lignin degradation rate has been described in Fig. 6. It was observed that the degree of lignin degradation attained was 65.3 % after 4 h of incubation. Subsequent lignin degradation was slow and reached 73.9 % after 8 h of incubation.

## Discussion

The yellow laccase obtained from *L. squarrosulus* (MR13) was purified using ammonium sulfate precipitation, ion-exchange chromatography on DEAE-cellulose followed by gel filtration chromatography. A laccase from monkey head mushroom *H. erinaceum* was purified up to a 15-fold purification factor. Its chromatographic steps included ion-exchange chromatography on DEAE-cellulose, CM-cellulose, Q-Sepharose and FPLC-gel filtration on Superdex 75 (Wang and Ng 2004). A blue laccase from *Ganoderma lucidum* was purified with a purification factor of 25.4-fold using DEAE-cellulose, Aff-gel blue gel, Con A-Sepharose and FPLC-gel filtration on Superdex 75 by Wang and Ng (2004). Similar yellow laccase from the fungi *Pleurotus ostreatus* D1 was purified from its crude solution by employing similar kind of purification steps (Pozdnyakova et al. 2006). In this present study 12.67-fold purification was achieved.

The concentrated purified enzyme solution was yellowish brown in colour. The absorption spectrum of purified laccase does not have the characteristic absorption at 610 nm, which is a typical characteristic of blue laccase for the presence of type 1 copper atom (Thurston 1994). These all sets of combinations were formerly pragmatic in yellow laccase of the fungi *P. tigrinus*, *Phlebia radiata* and *Phlebia tremellosa* (Leontievsky et al. 1997). The *A*_280_/*A*_610_ ratio of purified enzyme was found to be 312.5, which was higher than typical blue laccases (Leontievsky et al. 1997; Bezalel et al. 1996; Leonowicz and Crzywnowicz 1981; Laemmli 1970; Niku-Paavola et al. 1988).

*Lentinus squarrosulus* (MR13) yellow laccase appeared as a single band with a molecular weight of 66 kDa in SDS-PAGE. The molecular mass found was within the range of molecular masses for most of the fungal laccases reported (50–90 kDa) (Baldrian 2006). Laccases from *Albatrella dispansus* (62 kDa) (Wang and Ng 2006), *Coriolus zonatus* (60 kDa) (Koroljova et al. 1999), *H. echinacea* (63 kDa) (Wang and Ng 2004), *Marasmius quercophilus* (60 kDa) (Farnet et al. 2000) and *Trametes sanguinea* (62 kDa) (Nishizawa et al. 1995) have a molecular mass very close to that of *L. squarrosulus* (MR13) laccase. On the other hand, *P. eryngii* laccase has a small molecular mass of 34 kDa (Wang and Ng 2006), and a laccase from *Podosporaa anserina* has a large molecular mass of 383 kDa (Ng and Wang 2004).

Shin and Kim (1998) reported that the optimal pH of laccase from the fungus *Coriolus hirsutus* was 4.0. Solano et al. (1997) reported an optimum pH of 6.5 for melanogenic bacteria MMB-1. Niladevi et al. (2008) reported optimal pH for laccase from *Streptomyces psammoticus* as 8.5. These findings were similar to the present finding where the laccase enzyme showed significantly broad range of pH activity (pH 4.5–8.5) and pH stability (pH 4.5–9.0).

The presently studied yellow laccase was active over a wide range of temperatures. The typical optimum temperature ranges for laccases are 35–45 °C. Significant activity was detected at lower temperature of 25 °C and ~75 % of activity was maintained at 50 °C. The wide functional range is similar to that observed by Wang and Ng (2004) for the *Albatrella* enzyme. The laccase from *P. sanguineus* was more thermostable than expected for typical laccases (Palonen et al. 2003; Shin and Kim 1998; Thakker et al. 1992).

CD spectroscopic studies at different temperatures can be used to provide information on the stability of proteins. CD spectra of the yellow laccase supported the data that the enzyme maintained stability at the temperature range of 30–50 °C. During further analysis of the CD spectrum, it was observed that the yellow laccase showed two negative troughs at 30–50 °C, one of which is in the region of 220 nm and the other at 208 nm. This is considered to be a characteristic features for all α, β or α + β proteins with varying intensities related to the extent of secondary structural elements. With the further increase in temperature these features tend to change towards increase in random structure signifying structural breakdown and loss in activity of the enzyme.

The activity of the presently investigated yellow laccase was not greatly influenced by any of the metal ion studied at a concentration of 1 mM. Nagai et al. (2002) reported that *L. edodes* laccase was inhibited in the presence of 1 mM Ca^2+^ (70 %) and Zn^2+^ (64 %) and was enhanced by 40 % in the presence of 10 mM Cu^2+^. A laccase from *Coriolus versicolor* was strongly depressed by Ag^+^ (100 %), Al^3+^ (100 %), and Fe^3+^ (80 %), but was activated by Cu^2+^ (10 %) and Mg^2+^ (10 %) (Zhu and Ding 2003).

The enzyme was inhibited by all the inhibitors that were studied. However, the extent of inhibition varied greatly with the nature and concentration of the tested inhibitors. Sodium azide has been reported to prevent the substrate oxidation by laccase (Johannes and Majcherczyk 2000). The inhibitory effect of sodium azide on the *L. squarrosulus* was similar to other fungal laccases (Wu et al. 2011; Niladevi et al. 2008). EDTA, another well-known metal chelating agent fully inhibited the purified laccase at 2 and 5 mM concentrations. Similar results have also been reported from *S. cyaneus* (Arias et al. 2003). Most fungal laccases appear to be inhibited by this metal chelator (Baldrian 2006), although others, such as the laccase from *Pycnoporus sanguineus*, may be moderately resistant to EDTA (Lu et al. 2007). On the other hand, the inhibitory effect of thioglycolate on *L. squarrosulus* laccase is at par with previous reports for some other laccases (Niladevi et al. 2008; Sadhasivam et al. 2008).

The substrate specificity studies indicated that syringaldazine is the most suitable substrate for the presently studied yellow laccase (Table 6). Similar result has been reported by Calvo et al. (1998). Other methoxy-substituted compounds like ferulic acid and vanillic acid were not efficiently oxidized as compared to di-substituted compounds. These findings were in congruence with the previous reports (Palmieri et al. 1997). Among the substituted phenols such as catechol and resorcinol, the enzyme showed more affinity towards the ortho-substituted catechol than the meta-substituted resorcinol. Laccases have been reported to exhibit activity with para- and ortho-diphenols (Xu 1996) and very less reactivity has been observed generally with the meta-substituted phenols (Jolivalt et al. 1999).

In the presence of ABTS, the calculated *K*_m_ and *V*_max_ demonstrated the efficient oxidation ability of the yellow laccase toward the substrate. The *K*_m_ of presently studied yellow laccase was reletively lower than the previously mentioned reports. This feature implies that this yellow laccase have greater affinity towards the substrates. The *K*_m_ value of the laccases from *Trichoderma atroviride* and *Pycnoporus sanguineus* in the presence of the same substrate were determined to be 2,500 µM (Chakroun et al. 2010) and 77 µM (Lu et al. 2007), respectively. Laccase from *Chaetomium thermophilum* showed *V*_max_ value of 2.6 mmol min^−1^ mg^−1^ for the substrate ABTS (Chefetz et al. 1998). The values of the catalytic constants obtained for *L. squarrosulus* laccase were markedly higher than the above reported fungal laccases, which indicates the higher substrate specificity for laccase from *L. squarrosulus*.

In the present study it was found that,incubation time required for lignin degradation of *B. bamboos* was 6 h (Fig. 6) and further raise in incubation time does not demonstrate any significant effect. Lignin degradation ceased after attending upper limit, which might be due to the accumulation of lignin degradatory products which act as inhibitor for this yellow laccase. Incubation time for the present yellow laccase-mediated lignin degradation was shorter than any other reported values of enzyme mediated or whole cell mediated degradation of lignin or lignin model compounds (Cunha et al. 2010; Feng et al. 2010; Cho et al. 2009) but higher than the chemical or physical techniques (Yelle et al. 2008; Sun and Cheng 2007).

## Conclusion

The present study deals with the purification and characterization of a pH stable yellow laccase from a newly isolated fungus *L. squarrosulus* MR13. The three-step purification process was effective in concentrating and purifying yellow laccase with a significant yield of 12.67 %. Characterization studies showed that the enzyme possesses a molecular mass of 66 kDa, a pH optimum at 4.5 and 40 °C. It demonstrates activity towards a range of phenolic as well as non-phenolic compounds. The effect of different inhibitors on the purified yellow laccase conformed to the common model of laccase inhibition.
